# The need for biodiversity champions in psychiatry: the entwined crises of climate change and ecological collapse

**DOI:** 10.1192/bjb.2021.44

**Published:** 2021-08

**Authors:** Jacob Krzanowski

**Affiliations:** 1 South London and Maudsley NHS Foundation Trust, London, UK; 2 Royal College of Psychiatrists, London, UK

**Keywords:** Planetary health, psychiatry, public mental health, biodiversity, climate change

## Abstract

The past 20 years have seen the disappearance and degradation of biodiversity on earth at unprecedented rates. This phenomenon shares similar drivers to those behind climate change yet requires its own solutions. The twin catastrophes of climate change and biodiversity loss demonstrate how our health is bound up in the vitality of this planet. There has been an increasing effort on the part of healthcare professionals to appreciate this relationship, as evidenced by the growing influence of planetary health as a discipline. However, the health impacts of biodiversity loss have been less focused on than those brought on by climate change. Psychiatry's appreciation of the interface between environments and our health, alongside the evidence for the connection between nature and mental health specifically, prompt psychiatrists to ensure that the institution of healthcare throws its influence equally behind solutions to climate change as well as biodiversity loss.

This article is both a provocation and a call to action for psychiatry to champion biodiversity while raising awareness of the risk of ecological collapse. Although the connection may not at first be intuitive, our influence as healthcare professionals, appreciation for health as a concept defined by wellness rather than an absence of disease and understanding of how sick environments create illness, both seen and unseen, place responsibility firmly at our table to advance the cause of all life on earth.^1^

These arguments are underscored as international organisations such as the World Health Organization (WHO) increasingly recognise the causal links between our health and biodiversity loss.^2^ This is particularly important because framing the crisis through the lens of health may prove to be instrumental in shifting the tide towards the unified action needed.

In fact, new evidence is rapidly verifying that the loss of biodiversity in both quantity and quality has real impacts on our health, both mental and physical.^2,3^ This evidence is finally catching up with associations between nature and healthfulness that exist across many cultures, and the knowledge central to many indigenous cultures.^4^ , While this is an important development we should respect that gaps in our knowledge and apprehension regarding specific correlations should not preclude action.

We have been slow to respond to climate change and must bear this in mind. At the same time, in the face of uncertainty we should allow ourselves to be compelled by a change to life on earth described as the sixth planetary extinction, marked by a rate of extinction nearly 1000 times pre-human levels.^5,6^ It therefore falls to healthcare professionals to mitigate not only the consequences of climate change but also the equally concerning trend of ecological destruction.

## Climate change: one driver of biodiversity loss among many

The planetary crisis we are facing is more complicated and worse than was first imagined. For decades, scientists have understood that the planet is getting hotter.^7^ As the temperature rises, we are facing changes to the climate that are difficult to predict and harder to control. Climate change looks like rising water levels, protracted and intensified heat waves, changes to water composition and the degradation of ecosystems.^7^ These changes to the ‘weather’ are directly tied to increasing food and water scarcity, which in turn lead to displacement through forced migration and armed conflict.^8^

The consensus among those studying this phenomenon is that climate change is a product of changes to human behaviour since the industrial revolution.^9^ Through unregulated extraction and consumption of carbon-based fuels, the global temperature has steadily increased, long after this association was first suspected in 1896 by Swedish scientist Svante Arrhenius. The establishment of the Intergovernmental Panel on Climate Change (IPCC) by the United Nations in 1988 cemented a global commitment to better understand the development and implications of climate change.

As we grapple with the scale of the crisis, the need to unify climate change with social justice is increasingly underscored. People who have experienced and will continue to experience the greatest impacts of climate change on health are the least responsible for greenhouse gas emissions.^10^ To date, the vast proportion of total historical emissions has been produced by a proportionally small number of wealthy countries.^10^ However, in the past 20 years we are now coming to the sobering fact that climate change and its injustices are not all we must confront.

The year 2020 marked the end of a 10-year period designated the ‘UN decade on biodiversity’. The stated aim of the effort was to live in harmony with nature. This commitment was in part brought on by alarming reports such as the Millennium Ecosystem Assessment in 2005, which noted that humans had changed ecosystems more rapidly in the previous 50 years than at any point before.^11^

Biodiversity is a contraction of biological diversity and simply put is the stuff of life itself. The 2005 report defines biodiversity as variability among living organisms from all sources, including terrestrial, marine and other aquatic ecosystems, and the ecological complexes of which they are part.^11^ Biodiversity itself is the basis of the complex of plants, animals and microorganisms that make up an ecosystem.^11^ The accumulation of all of our planet's ecosystems is encapsulated by the biosphere.^12^

To help us understand our dependence on these systems the concept of ecosystem services was created. This model looks to explain all the ways in which we are connected in the web of life and outlines that ecosystems: (a) provide resources such as food, water, timber, medicines and fibre, (b) regulate climate, foods, disease, waste and water quality, (c) contribute to cultural experiences such as recreation, aesthetic enjoyment and spiritual fulfilment and (d) support soil formation, photosynthesis and nutrient cycling.^11^ The truth has been plainly described by the environmental activist Satish Kumar in the following stark terms: ‘We are nature. What we do to nature, we do to ourselves’.^13^

Since the mid-2000s, global efforts to address the collapse of biodiversity have emerged, most notably with the establishment of the Intergovernmental Science-Policy Platform on Biodiversity and Ecosystem Services (IPBES). Its 2019 Global Assessment Report describes a biosphere transformed and degraded by human activity, whereby ‘seventy-five per cent of the land surface is significantly altered, 66 per cent of the ocean area is experiencing increasing cumulative impacts, and over 85 per cent of wetlands (area) has been lost’.^14^ It identifies climate change as just one among several drivers – albeit one with a multiplying effect – that have an impact on both nature and human well-being, alongside habitat change, invasive alien species, overexploitation and pollution.^11,14^ The thing that cannot be forgotten is that many of these losses, in particular of species and biodiversity, are irreversible.^15^

The pivotal importance of attending to the loss of biodiversity is reflected in its inclusion within the UN's sustainable development goals and the UN's choice to designate the 2020s as the ‘decade for eco-system restoration’.^16,17^ Biodiversity loss and climate change share human behaviour as their root cause, but while their solutions are mutually beneficial, they demand distinct strategies. The centrality of biodiversity to the survival of all life, coupled with its demand for solutions apart from ones that address sustainability or climate change per se, is why biodiversity loss specifically requires champions among healthcare professionals.

## Planetary health: an opportunity for healthcare's growing role

Why has change been so slow to come? Resistance has been in large part cultivated by corporations, politicians and the interests of the wealth that entwines them.^18^ In our own community, David Pencheon, a former head of the NHS Sustainable Development Unit, has referenced a kind of ‘moral offset’ that means health professionals feel less motivated to act on the climate crisis because their work already improves lives.^19^ Health services also prioritise acute illness over prevention, discounting future risk in favour of short-term medical responsibility.^19^

At the start of the millennium, the importance of an interdisciplinary approach to health and climate change was recognised. Institutions such as the *Lancet* sounded the need to frame the crisis in terms of its effects on health. Although not a new term at the time, the concept of planetary health was fully endorsed in 2015 by a joint effort between the *Lancet* and the Rockefeller Foundation, a sign that healthcare as an institution had begun to throw its weight behind a response to the planetary crisis.^20^

Planetary health in the simplest sense makes the assertion that human health is inextricably linked to the health of the planet. It can be understood as a discipline that borrows from public and environmental health but is bold and explicitly political. Richard Horton, editor-in-chief of the *Lancet*, described planetary health in 2018 as ‘an inquiry into our total world. The unity of life and the forces that shape those lives’.^21^

At the heart of planetary health lies a paradox that many of the gains to health have come from an untold exploitation of the environment.^22^ The field does not shy away from this uncomfortable fact and indeed is based on the belief that a more balanced relationship wherein both humans and the planet thrive is possible and essential. It suggests that acting as stewards of the biosphere, humans can move from an exploitive to a nurturing role.^22^ As a whole, the discipline and its advocates seek to bring the influence of healthcare, as an institution, into the centre of discussions and campaigning around the climate and ecological crises. Such aspirations are in line with the increasing anticipation that casting planetary change through the lens of our own health may be instrumental in helping to inspire the type of action urgently needed.^23^

Alongside this developing approach, in October 2020 the National Health Service (NHS) made the impressive commitment to become the first carbon-neutral national health service.^24^ Yet the report leaves the issue of biodiversity loss behind. This trend towards focusing on climate change is similarly reflected in the *Lancet*'s countdown on health and climate change,^25^ and as of February 2021 four NHS trusts have declared climate emergencies since Newcastle upon Tyne Hospitals NHS Foundation Trust first did so in July 2019.

Although the field of planetary health is premised on a connection between health and nature, clinicians are only beginning to grapple with its implications. This may reflect the fact that the impacts of biodiversity loss on humans are dizzying in their scope but also, with respect to mental health particularly, can be harder to understand and therefore measure. Moreover, although the emphasis on climate change has created a focus point for action, it unintentionally creates a conceptual blind spot whereby the broader threat of ecological collapse can feel like an ‘add-on’ and therefore harder to address or even a distraction.

## Biodiversity and human health

The concept of ecosystem services underlines how essential biodiversity is to life on earth. Without pollinators many plants cannot reproduce and without plants no oxygen is made. Links between health and biodiversity have been clearly drawn and are now continuously emerging in greater breadth and resolution.^2^ In 2020, the COVID-19 pandemic highlighted the connection between biodiversity loss and infectious disease, while research elsewhere highlighted the role of ecosystems in sequestering carbon.^26,27^ Moreover, a growing evidence base recognises that our well-being and mental health are tied to the health of our ecosystems. Similarly, the destruction and loss of natural spaces is increasingly shown to affect our mental, emotional and spiritual health.

### Carbon capture

The links between climate change and biodiversity are increasingly clear. Rising global temperatures degrade our richest ecosystems, yet these also represent essential storages of carbon. A recent paper by Dinerstein et al states that nearly all of the remaining native ecosystems will require preservation to keep the global average temperature rise below 1.5°C.^27^ The paper brings together startling facts, including that intact forests sequester twice as much carbon as planted monocultures, and highlights that diverse systems from peatlands to mangroves are also important carbon storehouses. They make clear that it is the rich biodiversity of these natural spaces that allows carbon to be stored in such amounts. This understanding motions at the need to consider climate change and biodiversity loss in the same breath with efforts to ‘pair nature and climate deals which are mutually reinforcing’.^27^ We are in a race against time where every piece of the puzzle is connected for better and worse.

### Pandemics

Pandemics act on our physical and mental health through both direct and indirect mechanisms resulting in immediate but also far-reaching changes to individuals and societies. The origins of COVID-19 and its development into a global pandemic are closely tied to ecological destruction.^26^ It is a double tragedy that the experience of lock-downs and quarantine have highlighted the importance of natural spaces for many.^28^ Concerns about the impact of biodiversity loss were made as early as 2005, when Paul Epstein noted that ‘widening social inequalities and changes in biodiversity have apparently contributed to the resurgence of infectious diseases.’^29^ The 2020 IPBES workshop on biodiversity and pandemics is clear about the drivers behind COVID-19, stating that ‘pandemics have their origins in diverse microbes carried by animal reservoirs, but their emergence is entirely driven by human activity […] The underlying causes of pandemics are the same global environmental changes that drive biodiversity loss and climate change’.^26^

More sobering is the warning that, without changing these underlying drivers, we can expect more frequent pandemics. Indeed, there are an estimated 1.7 million undiscovered viruses, 631 000–827 000 of which could have the ability to infect humans. Averting further pandemics rests on a reversal of the unsustainable exploitation of the environment driven by demand from wealthier countries and emerging economies.^26^

### Nature and well-being

There is a growing recognition of the impact of nature on well-being.^30^ Exactly how exposure to nature benefits humans is not entirely clear. Theories such as that proposed by Kaplan & Kaplan in 1989 suggest that exposure to nature acts on well-being by modulating stress through restoration of our attention.^31^ Nature is also believed to enhance our well-being by supporting health-promoting activities such as physical activity and social interaction.^30,32^ Astonishingly, from the view of public health, green spaces have also been found to be equigenic, a term referring to interventions that disrupt the normal health disparities arising from socioeconomic inequality.^33^ In urban environments, such spaces provide further mental health benefits as they mitigate heat islands, improve air quality and prevent floods, leading to longer-term and more holistic health benefits.^34^

Crucially for psychiatrists, links have also been made between natural spaces of high value and improved mental well-being.^3,32,34^ A 2014 literature review by Lovell et al, however, offers caution about drawing definitive associations between increased biodiversity and health and encourages further research on this relationship.^35^ Recently, a study of the impact of biodiversity across Europe added the finding that the diversity of birds in an ecosystem improved people's life satisfaction.^36^ Irvine et al, in a review of spirituality and biodiversity, offer that ‘there is suggestive evidence that biodiversity appears to contribute to spiritual outcomes’.^37^ They note that these sorts of observation are critical in making clearer links between conservation of biodiversity and human well-being.

### Eco-distress and solastalgia

During the 2019–2020 Australian bushfire season, it was estimated that 3 billion animals were affected.^38^ This is an unspeakable tragedy for the life lost and those living in the areas affected by the fires. However, it was also witnessed by many abroad, eliciting feelings of helplessness, confusion, guilt, grief and anxiety. Much like the loss of these ecosystems, it is unclear how the growing presence of such emotions will affect people. An increasing interest in such questions has been seen with the emergence of concepts such as eco-distress and solastalgia.^39^ For the moment, these constructs do not describe mental illness but rather proportional reactions to traumatic ecological events. The term solastalgia specifically describes the distress resulting from the transformation and degradation of one's home environment. Although such concepts are relatively new, they underscore the role that mental health professionals play in raising awareness about ecological collapse and its psychological toll.

## How to respond?

Recognising biodiversity loss, and ecological collapse more broadly, does not necessarily mean dividing attention from climate change. Indeed, one of the best ways healthcare systems can address ecological collapse is by mitigating their greenhouse gas output: if they were a nation, healthcare systems globally would constitute the fifth largest greenhouse gas emitter.^40^ What is key is understanding the crisis holistically, so that psychiatrists work against the conceptual fragmentation that prevents bold systemic solutions.

Within mental health services, pharmaceuticals contribute around 20% of our total carbon footprint.^41^ The development and adoption of sustainably informed prescribing practices provide a concrete way for institutions and individual clinicians to play their part.^42^ Sustainable prescribing would need to take into account the environmental implications of common prescribing practices, including polypharmacy, unclear durations of treatment and exceeding recommended dose ranges.^43^ Such considerations would create greater space to offer non-medication-based complementary therapies, including those that are nature-based.

Beyond the ethics of accurately representing the broader ecological crisis, this holistic approach also makes it more likely that policies that tackle greenhouse gas emissions alongside other drivers of ecological collapse will be found and implemented. For example, a green rooftop developed by a hospital to reduce air conditioning could also then be cultivated with plants favoured by local pollinating insects. If tended or enjoyed by psychiatric in-patients, this becomes an intervention in which climate change, biodiversity and therapeutic impacts are married.

Part of the difficulty in knowing how psychiatrists should most effectively lend their voices to the crisis of biodiversity loss is the immensity that the term implies. How do we go about saving the biosphere? The Global Deal for Nature proposes a firm target of protecting 30% of the earth by 2030, which lays the ground for global action.^27^ This clearly stated goal, much like the limit of 1.5°C warming for climate change, helps us to believe that, in spite of overwhelming complexity, conceptually simple strategies such as conservation can work.

With this as a foundation, psychiatrists could focus on two kinds of strategic action. First, psychiatrists should support and initiate further research into the relationship between mental health and the natural world. In the UK, this effort could be advanced by innovative collaborations between mental health institutions and wildlife organisations such as Natural England and the Wildlife Trusts. Psychiatrists should also engage with knowledge and practices developed by colleagues in the field of ecopsychology, including giving consideration to concepts such as nature connectedness.^44^ Supported by a more robust evidence base, psychiatrists will better identify ecology-related causal factors in mental illness, as well as potential avenues of support. This sensitivity, in turn, will increase patient awareness of how mental health is entwined with natural spaces.

In adopting the cause of biodiversity, new research would be complemented by expanded clinical experiences and opportunities for first-hand observation of the nature–mental health interface. Psychiatrists, for example, can help make more immediate changes to the environments of our health services. This includes championing public green spaces and supporting trusts and hospitals to tend their own natural spaces. NHS Forest, for example, is a programme that has promoted planting trees on NHS grounds.^45^ The conservation of areas close to health services more easily allows for the integration of sustainable green care options within mental healthcare. A project such the Green Walking initiative, which has seen eight trusts introduce green walking programmes for psychiatric in-patients, shows how easily local green spaces can be integrated into clinical care and generates insights into best practice that complement ongoing research.^46^

The second kind of action would see psychiatrists advocate, campaign and highlight the mental health implications of ecological collapse in their organisations and communities in a way that bridges people's lived experiences with the aspirational scale of planetary health.

The American Psychological Association has made recommendations for how mental health professionals can highlight the relationship between health and climate change, identifying education, awareness, communication and motivating climate solutions as important areas of action.^47^

Although these areas are humble it should be remembered that healthcare's involvement in campaigning against climate change began with the premise that simply sharing information can lead to a change in perspective. Developments such as the NHS net-zero plan, the establishment of planetary health institutes around the world, publications focused on planetary health, the *Lancet* countdown and the UK Health Alliance on Climate Change (UKHACC) all emerged as a result of passionate voices calling for action.

Speaking eloquently and listening carefully are skills that mental health professionals use every day. As leaders within mental health, psychiatrists should speak to the government and the health sector broadly to ensure that the relationship between biodiversity and health is heard. Advocating for the inclusion of instruments that take into consideration the impact on mental health of developments and changes to land usage, for example, would be a clear and effective demand. In summary, all of these suggested actions offer important steps in conceiving of a sustainable mental health service that champions a more preventive style of patient care.

## Conclusions

At the heart of psychiatry is the appreciation that our health and experience are influenced by a complex web of interactions. Carrying this knowledge has allowed psychiatrists to make bold observations on the very real impact of social inequality on people's mental health.^48^ This ability to understand how influenced people are by their environments is also why psychiatrists should see the growing threat to nature as entwined with climate change and ultimately with mental and physical well-being for everyone.

## Data Availability

Data availability is not applicable to this article as no new data were created or analysed in this study.

## About the author

**Jacob Krzanowski** is a specialist registrar in general adult psychiatry with South London and Maudsley NHS Foundation Trust, an Associate Registrar in Sustainability for the Royal College of Psychiatrists, London, and an Associate of the Centre for Sustainable Healthcare, Oxford, UK.
